# Diagnostic Accuracy of Delayed Phase Post Contrast Computed Tomographic Images in the Diagnosis of Focal Liver Lesions in Dogs: 69 Cases

**DOI:** 10.3389/fvets.2021.611556

**Published:** 2021-03-04

**Authors:** Silvia Burti, Alessandro Zotti, Federico Bonsembiante, Barbara Contiero, Tommaso Banzato

**Affiliations:** ^1^Department of Animal Medicine, Productions and Health, University of Padua, Padua, Italy; ^2^Department of Comparative Biomedicine and Food Science, University of Padua, Padua, Italy

**Keywords:** decision tree, HCC (hepatic cellular carcinoma), contrast - enhanced CT, computed tomography, focal liver lesion

## Abstract

To describe the computed tomographic (CT) features of focal liver lesions (FLLs) in dogs, that could enable predicting lesion histotype. Dogs diagnosed with FLLs through both CT and cytopathology and/or histopathology were retrospectively collected. Ten qualitative and 6 quantitative CT features have been described for each case. Lastly, a machine learning-based decision tree was developed to predict the lesion histotype. Four categories of FLLs - hepatocellular carcinoma (HCC, *n* = 13), nodular hyperplasia (NH, *n* = 19), other benign lesions (OBL, *n* = 18), and other malignant lesions (OML, *n* = 19) - were evaluated in 69 dogs. Five of the observed qualitative CT features resulted to be statistically significant in the distinction between the 4 categories: surface, appearance, lymph-node appearance, capsule formation, and homogeneity of contrast medium distribution. Three of the observed quantitative CT features were significantly different between the 4 categories: the Hounsfield Units (HU) of the radiologically normal liver parenchyma during the pre-contrast scan, the maximum dimension, and the ellipsoid volume of the lesion. Using the machine learning-based decision tree, it was possible to correctly classify NHs, OBLs, HCCs, and OMLs with an accuracy of 0.74, 0.88, 0.87, and 0.75, respectively. The developed decision tree could be an easy-to-use tool to predict the histotype of different FLLs in dogs. Cytology and histology are necessary to obtain the final diagnosis of the lesions.

## Introduction

Focal liver lesions (FLLs) are common in dogs, especially in older patients. Malignant primary hepatic tumors represent <1.5% of all the malignant tumors in dog. More than 50% of malignant FLLs in dogs are hepatocellular carcinomas (HCC), whereas bile duct carcinomas (BDC) account for 22–40% of cases. Metastatic tumors (from pancreatic, splenic and gastroenteric tract in most cases) involving the liver are 2.5 times more frequent than primary tumors (1–3). Benign FLLs, especially in older dogs, are mostly nodular hyperplasia (NH), hepatocellular adenoma, and bile duct adenoma (1, 4).

In human medicine the computed tomographic (CT) features of different FLLs in the arterial, portal and delayed phase are well-described, and, therefore, it is possible to infer the histopathologic subtype of a FLL from its CT features (5–7). For example, the presence of a hypervascular pattern in a heterogeneous enhancing hepatic lesion during the arterial phase, is a feature often associated with HCCs (6).

In human medicine, the increasing availability of triple-phase CT, magnetic resonance imaging (MRI) and positron emission tomography (PET) have improved the scope to detect and diagnose FLLs (8). Moreover, the etiopathology of HCCs is well-known (8), and the diagnostic and therapeutical approaches to this tumor are well-detailed and based on the appearance of the lesions during the imaging examinations and on the histotype of the lesions (8, 9). In animals, the etiopathology of FLLs is less detailed compared to human medicine, studies have involved a lower number of cases, and the inclusion and exclusion criteria related to the features of the lesions (e.g., margins, number, dimension) are less restrictive.

The CT features of FLLs in dogs have seldom been described (4, 10–13). The studies on this topic are very heterogenous, indeed, a variety of features, scanning protocols, lesions, were evaluated in the different studies. A moderate to high accuracy of some CT features [e.g., delayed phase enhancement (13, 14)], in the distinction between benign and malignant masses is reported. Despite such encouraging results previous studies have only considered either only benign or malignant lesions (13, 14), or only certain lesion histotypes (4, 10, 11). Another important limitation is that, to date, most of the studies have only evaluated the accuracy of individual features and an algorithm to classify hepatic lesions based on their CT features has not been developed yet. In such a scenario, histopathology is still the gold-standard method for characterization of FLLs in dogs.

The possible applications of machine learning algorithms have been widely explored in human diagnostic imaging in the last decades (15). On the other hand, in the last decade, the possibilities offered by this technology has raised an increasing interest also in veterinary medicine (16–20). Machine learning comprise a wide range of algorithms that can be broadly divided into machine learning and deep learning (21). Generally speaking, the main difference between the two systems is that machine learning algorithms are statistical methods that are applied on human crafted features extracted from the images whereas deep learning algorithms, are end-to-end algorithms capable of automatically extracting the features from the images and analyzing them accordingly.

The aims of the present study are: (1) to describe the quantitative and qualitative computed tomographic features in pre-contrast and in the delayed phase of different histopathological subtypes of FLLs in dogs; (2) to develop a machine learning-based decision tree to assist the radiologist and the clinician in predicting different histopathological subtypes of FLLs based on their CT features. We hypothesized that a diagnostic algorithm, based on the CT features as described by the radiologist, could help in the prediction of the histopathological type of FLL.

## Materials and Methods

### Study Population

Dogs referred to the Pedrani Veterinary Clinic (Via Caldierino 14, Zugliano, Vicenza, Italy) and to the Veterinary Teaching Hospital of the University of Padua (Viale Dell'Università 16, Legnaro, Padua, Italy) between June 2015 and January 2020 and which underwent computed tomographic examination and had FLL diagnosed with cytopathology and/or histopathology were retrospectively selected. Complete signalment was recorded for each patient. Dogs with hepatic masses/nodules not diagnosed on pathology, or which underwent chemotherapy before the tomographic examination, were excluded.

All the methods were carried out in accordance with the relevant guidelines and regulations. This study was conducted respecting the Italian law D. Leg.vo 26/2014 (that transposes EU Directive 2010/63/EU). Nevertheless, since the data used in this study were part of routine clinical activity, no ethical committee approval was required. Informed consent regarding the treatment of personal data was obtained from the owners.

### Cytopathological and Histopathological Examination

Cytological samples were obtained through ultrasound-guided fine-needle aspiration of the hepatic mass. Twenty-one Gauge needles were always used. Aspirates were spread on glass slides that were air-dried, stained with May-Grünwald-Giemsa stain and cover-slipped. The evaluation of the cytological slides was performed by one cytologist (FB).

Histological samples were obtained through ultrasound-guided Tru-cut biopsy of the hepatic mass. Tissue samples were fixed in 10% neutral formalin, processed by dehydration in a graded ethanol series and embedded in paraffin. Histological examination was carried out on 4-μm-thick sections stained with haematoxylin and eosin by one pathologist.

### Computed Tomography Examination

All the animals were fasted for a 12-h-period prior to examination. General anesthesia was always administered. The CT examinations were performed using three different scanners (Asteion super4, Toshiba Medical System Corporation; Revolution ACT, General Electric Medical System; Optima CT 520 Series, General Electric Medical System). Due to the different technology of the scanners, slightly different scanning protocols were used. The scanning protocols were as follows: In both facilities contrast medium (Ioversol 350 mg/ml, Optiray 350, LIEBEL-FLARSHEIM COMPANY LLC, USA) at the dosage of 660 mg I/kg of body weight was injected through an IV catheter placed in the cephalic vein. At the Pedrani Veterinary Clinic the contrast medium was administered by means of an injector. At the Veterinary Teaching Hospital, the contrast medium was manually administered through an intravenous bolus at the fastest possible rate. In both institutions a standard total-body scan with a pre-contrast and a delayed phase, starting from the nose tip at 60–70 s after the end of contrast medium injection, was used. This means that, considering the post-start injection-scanning at the liver site a delay ranging from 69 to 105 s at the Pedrani Veterinary Clinic and a 74–120 s at the Veterinary teaching Hospital should be considered. All the patients were placed on ventral recumbency during the scan.

All the images were stored as digital imaging and communication in medicine (DICOM) files.

### Image Analysis

All the scans were reviewed by two experienced radiologists (AZ and SB) using a picture archiving and communication system (PACS) workstation (RadiAnt DICOM Viewer 5.5.0). The qualitative and quantitative CT features were evaluated during both the pre-contrast phase and the delayed phase. In the case of multiple lesions, only the CT features of the lesions that were sampled have been described. All the studies were displayed in a soft tissue window (WW: 400 HU– WL: 40 HU).

The following qualitative features were evaluated: (1) margins (well- or ill-defined); (2) surface (regular or irregular); (3) appearance (solid or cyst-like) - the lesion was classified as “cyst-like” if at least one area having a measured Hounsfield Unit (HU) value similar to that of the gallbladder of the same animal (representing possible necrosis or hemorrhage), was present (10); (4) portal lymph-nodes appearance (normal or abnormal) – portal lymph nodes were graded as abnormal if any of the following changes were evident: (a) lymphoadenomagaly (b) heterogeneous (c) irregular shape; (5) capsule formation (present or absent) - the presence of a capsule was reported if a thin and hyperenhancing fibrous peripheral border, encompassing most of the lesion, was present and care was placed to differentiate between real capsule formation and the presence of enlarged vessels or sinusoids mimicking the presence of a real capsule (22); (6) portal invasion, meaning the invasion of the portal vein and its branches (present or absent); (7) homogeneity in the distribution of the contrast medium inside the lesion (homogeneous or heterogeneous); (8) enhancement pattern (prevalently central, marginal, or diffuse distribution).

The following quantitative characteristics were evaluated: (1) attenuation (measured as an HU value) of the radiographically normal liver parenchyma surrounding the lesion, both in pre- and post-contrast scans; (2) attenuation (HU value) of the lesion both in pre- and post-contrast scans; (3) maximum transverse diameter; (4) volume - the shape of the lesion was considered to be an ellipsoid and the formula $V=\frac{4}{3}\pi \;\left(height^{*}width^{*}length\right)$ was applied (23); (5) attenuation of the lesion compared to that of the radiologically normal liver parenchyma in the pre-contrast images (hypoattenuating, isoattenuating, or hyperattenuating); (6) enhancement degree of the lesion compared to that of the radiologically normal liver parenchyma in post-contrast images (hypoenhancing, isoenhancing, or hyperenhancing). The attenuation and the enhancement degree of the lesion were determined based on the difference between the HU value measured in the lesion and the HU value measured on the radiologically normal liver parenchyma. If the difference between the lesion and the parenchyma felt in the ± 10 HU range, the lesion was classified as iso-attenuating/enhancing; with a difference > + 10 HU the lesion was classified as hyper-attenuating/enhancing; if the difference was lower than −10 HU, the lesion was classified as hypo-attenuating/enhancing (11, 12). The CT features were evaluated by two of the authors of this study (SB: with 4 years of experience in diagnostic imaging and AZ: with 20 years of experience in diagnostic imaging) that were blinded to the results of the histopathological examination.

HU values were measured on the three circular regions of interest (ROIs) which could be placed in different regions of both normal and pathological parenchyma. The same ROIs were placed in pre- and post-contrast images.

### Statistical Analysis

All the statistical analysis was performed using R-software version 3.6.1 (24). The difference in the distribution of the qualitative variables was analyzed with the chi-Square test (χ^2^) or with Fisher's exact method. *Post-hoc* multiple comparisons among levels were performed using Marascuilo approach. Differences in the distribution of the quantitative variables were analyzed with a one-way analysis of variance (ANOVA) for normally distributed data, or with the Kruskal-Wallis test for non-normally distributed data. The Tukey-Kramer method was used for multiple comparison tests after ANOVA analysis. A Steel-Dwass-Critchlow-Fligner procedure was used for pairwise comparison testing after Kruskal-Wallis analysis. A *p* < 0.05 was considered as statistically significant.

A machine learning-classification tree analysis was performed to detect the best discriminating CT features. A recursive partitioning method was adopted using the rpart package of R (25). This package builds a decision tree based on a three-step procedure. In the first step, the feature that provides the best splitting of the data into two groups is selected. The second step of the procedure uses a 10-fold cross-validation to select the tree having both the lowest number of branches and the lowest misclassification rate. Thereafter, the developed tree is applied to the original dataset and sensibility, specificity, accuracy and misclassification rate are calculated.

## Results

### Patients

Based on the inclusion criteria, 69 dogs of different breeds (37 females and 32 males, with mean age of 11 years ranging from 4 to 16.5 years), with pathologically diagnosed FLLs, and which underwent a CT examination, were included. Cytopathology was performed in 54 dogs. Tru-cut biopsy was performed in 13 dogs, and both cytology and histology were performed in 3 dogs. In two of these latter cases there was an agreement between cytology and histology (both were suggestive of HCC); in one case the cytological diagnosis was blood collection and vacuolar degeneration, while NH was diagnosed by means of histology. Benign lesions were diagnosed in 37 cases (1 biliary duct adenoma, 1 haematoma, 1 inflammation, 2 hepatocellular adenomas, 2 normal liver parenchyma, 11 degenerations, and 19 nodular hyperplasia), and malignant lesions were diagnosed in 32 cases (1 mast cell tumor, 1 plasmocytoma, 1 biliary duct carcinoma, 1 undifferentiated carcinoma, 1 melanoma, 1 metastasis of mammary neoplasia, 2 lymphomas, 4 endocrine neoplasia, 7 sarcomas and 13 HCCs).

Due to the large variability in the histological subtypes of the lesions included in the study, the patients were grouped into the following four categories for the statistical analysis: NHs (19 cases); OBLs (18 cases), HCCs (13 cases), and OMLs (19 cases).

### Image Analysis

A summary of all the CT parameters evaluated, along with the *p*-values of the statistical tests, is reported in Table 1–(qualitative features) and Table 2 (quantitative features).

**Table 1:** Number of cases, classified based on cytological or histological examination, showing the qualitative features, along with the *p*-value.

	**Category**				
	**Nodular hyperplasia** ***(n = 19)***	**Other benign lesions^†^** ***(n = 18)***	**Hepatocarcinoma** ***(n = 13)***	**Other malignant lesions^‡^** ***(n = 19)***	***p*-value**
Well-defined margins	14 (73.7%)	13 (72.2%)	13 (100%)	11 (57.9%)	0.07
Irregular surface	14 (73.7%)^ab^	7 (38.9%)^b^	13 (100%)^a^	17 (89.5%)^a^	<0.01
Abnormal lymph nodes	5 (26.3%)^ab^	1 (5.6%)^b^	7 (53.8%)^a^	11 (57.9%)^a^	<0.01
Presence of portal invasion	0	0	0	2 (10.5%)	0.39
Presence of capsule formation	0^b^	7 (38.9%)^a^	7 (53.8%)^a^	9 (47.4%)^a^	0.03
Cyst-like appearance	11 (57.9%)^ab^	9 (50.0%)^b^	12 (92.3%)^a^	10 (52.6%)^a^	0.03
Heterogeneity post-contrast medium	12 (63.2%)^b^	6 (33.3%)^b^	13 (100%)^a^	13 (68.4%)^b^	<0.01
Hypoattenuation pre-contrast medium	13 (68.4%)	15 (83.3%)	12 (92.3%)	16 (84.2%)	0.53
Hypoenhancement post-contrast medium	15 (78.9%)	16 (88.9%)	13 (100%)	17 (89.5%)	0.65
Diffuse enhancement pattern	16 (84.2%)	14 (77.8%)	11 (84.6%)	15 (78.9%)	0.7

† *other benign lesions = 1 biliary duct adenoma, 1 inflammation, 1 haematoma, 2 adenomas, 2 normal parenchyma, 11 degenerations*.

‡ *other malignant lesions = 1 mast cell tumor, 1 plasmocytoma, 1 biliary duct carcinoma, 1 undifferentiated carcinoma, 1 melanoma, 1 metastasis of mammary neoplasia, 2 lymphomas, 4 endocrine neoplasia, 7 sarcomas*.

*Different letters along rows means values significantly different for post-hoc multiple comparisons with Marascuilo approach*.

**Table 2:** Quantitative features of the lesions, classified based on cytological or histological examination, are reported as medians along with the first and third quartile values and the *p*-value.

	**HU normal liver pre-CE**	**HU normal liver post-CE**	**HU lesion pre-CE**	**HU lesion post-CE**	Max dimension^*^	Ellipsoid volume^**^
**DIAGNOSIS**						
Nodular hyperplasia (*n* = 19)	63.82 (53.79–69.79)^ab^	144.54 (120.59–169.15)	45.68 (40.72–54.79)	114.37 (50.96–144.87)	4.53 (2.45–6.75)^ab^	40.78 (6.15–112.86)^ab^
Other benign lesions^†^ (*n* = 18)	66.84 (64.36–72.54)^a^	137.60 (126.71–154.01)	39.50 (29.94–45.99)	75.65 (61.37–121.17)	2.15 (1.12–5.33)^b^	2.41 (0.39–26.78)^c^
Hepatocarcinoma (*n* = 13)	58.63 (53.12–63.02)^b^	127.72 (116.12–135.06)	41.48 (34.87–46.93)	67.39 (56.03–83.93)	11.11 (5.67–13.76)^a^	393.57 (54.80–727.31)^a^
Other malignant lesions (*n* = 18)	60.03 (54.59–64.55)^b^	142.87 (117.56–157.18)	39.93 (34.39–46.12)	83.19 (66.32–121.40)	3.59 (2.11–4.61)^b^	8.31 (3.67–23.60)^bc^
*p*-value	<0.01	0.29	0.80	0.13	<0.01	<0.01

*HU, Hounsfield Unit; c.m., contrast medium*.

* *Values are expressed in cm*.

** *Values are expressed in cm^3^*.

Different letters along columns means values significantly different for post-hoc multiple comparisons.

† *Other begin lesions = 1 biliary duct adenoma, 1 inflammation, 2 haematoma, 2 adenomas, 2 normal parenchyma, 11 degenerations*.

*‡Other malignant lesions = 1 mast cell tumor, 1 plasmocytoma, 1 biliary duct carcinoma, 1 undifferentiated carcinoma, 1 melanoma, 1 metastasis of mammary neoplasia, 2 lymphomas, 4 endocrine neoplasia, 7 sarcomas*.

Among the qualitative features the surface (χ^2^ = 19.80; *p* < 0.01), appearance (χ^2^ = 8.75; *p* = 0.03), lymph-node appearance (χ^2^ = 13.19; *p* < 0.01), capsule formation (χ^2^ = 9.23; *p* = 0.03), and homogeneity in the distribution of the contrast medium inside the lesion (χ^2^ = 13.79; *p* < 0.01) showed statistically significant differences. No significant differences were evident for the characteristics of the margins (χ^2^ = 7.14; *p* = 0.07), the presence of portal invasion (χ^2^ = 3.02; *p* = 0.39), the enhancement pattern (χ^2^ = 3.83; *p* = 0.70), attenuation during the pre-contrast phase (χ^2^ = 2.21; *p* = 0.53), and enhancement during the post-contrast phase (χ^2^ = 4.19; *p* = 0.65). Most of the HCCs included in this study (>90%), showed an irregular surface, a cyst-like appearance and heterogeneity post-contrast medium. The presence of abnormal lymph-nodes was equally recorded both in HCC and OML (more than 50% of the cases). The presence of a capsule was recorded in all lesions, except NH.

All the quantitative variables showed non-normal distribution. Therefore, values are reported as medians along with the first and third quartiles, and differences were calculated using the Kruskal- Wallis test. As a result of the Kruskal-Wallis test, the HU value of the radiologically normal liver parenchyma during the pre-contrast phase (*K* = 12.71; *p* < 0.01), maximum dimension (*K* = 14.60; *p* < 0.01) and ellipsoid volume (*K* = 18.21; *p* < 0.01) showed statistically significant differences. *Post-hoc* testing revealed the following significant differences: [1] between HCCs and OBLs regarding maximum dimension (*p* < 0.01), ellipsoid volume (*p* < 0.01) and the HU value of the radiologically normal liver parenchyma during the pre-contrast phase (*p* < 0.01); [2] between HCCs and OMLs regarding maximum dimension (*p* = 0.03), and ellipsoid volume (*p* < 0.01); [3] between nodular hyperplasia and OBLs (*p* = 0.04); [4] between other malignant and OBLs for the HU value of the radiologically normal liver parenchyma during the pre-contrast phase (*p* < 0.01).

No statistically significant differences were evident for the remaining quantitative features: the HU value of the radiologically normal liver parenchyma during the post-contrast phase (*K* = 3.80; *p* = 0.29), and the HU value of the lesions during both pre-contrast (*K* = 2.76; *p* = 0.43) and post-contrast (*K* = 4.93; *p* = 0.18) phases.

NH is a benign condition, which is often an occasional finding, but it could be a diagnostic challenge in a patient with a known history for another malignancy (22). In the present study, NHs were mainly hypoattenuating (68.4%) and hypoenhancing (78.9%) lesions showing a diffuse contrast enhancement pattern (84.2%), with homogeneous distribution (63.2%), well-defined margins (73.7%), an irregular surface (73.7%) and a cyst-like appearance (57.9%). The hepatic lymph nodes were radiologically normal in 14 out of 19 cases (73.7%), whereas neither portal vein invasion nor a real capsule formation were evident in any case.

The OBLs were mainly hypoattenuating (83.3%) and hypoenhancing (88.9%) lesions characterized by a diffuse enhancement pattern (77.8%), homogeneous distribution (66.7%), well-defined margins (72.2%), a regular surface (61.1%), and with both a cyst-like (50%) and solid (50%) appearance. Hepatic lymph nodes were almost always radiologically normal (17/18), and portal vein invasion was never detected. A fibrous capsule was evident in 7 out of 18 patients.

HCCs were mostly cyst-like (12/13, 92.3%), hypoattenuating (12/13) and hypoenhancing lesions (100%), with a diffuse enhancement pattern (84.6%), a heterogeneous distribution (100%), well-defined margins (100%), and an irregular surface (100%). The hepatic lymph nodes were abnormal in 53.8% of the cases (7/13), and portal vein invasion was never evident. The lesions were surrounded by a fibrous capsule in 53.8% of cases. *Post-hoc* tests revealed no significant differences between HCCs and NHs for both qualitative variables and quantitative variables.

The OMLs were mainly hypoattenuating (84.2%) and hypoenhancing (89.5%), showing a diffuse enhancement pattern (78.9%), heterogeneous distribution (68.4%), well-defined margins (57.9%) and an irregular surface (89.5%). They had both a cyst-like (52.6%) and a solid (47.4%) appearance. The hepatic lymph nodes were abnormal in 57.9% of cases. Portal vein invasion was evident only in 2 patients (10.5%). Fibrous capsule formation was evident in 9 out of 19 (47.4%).

**Figure 1:**
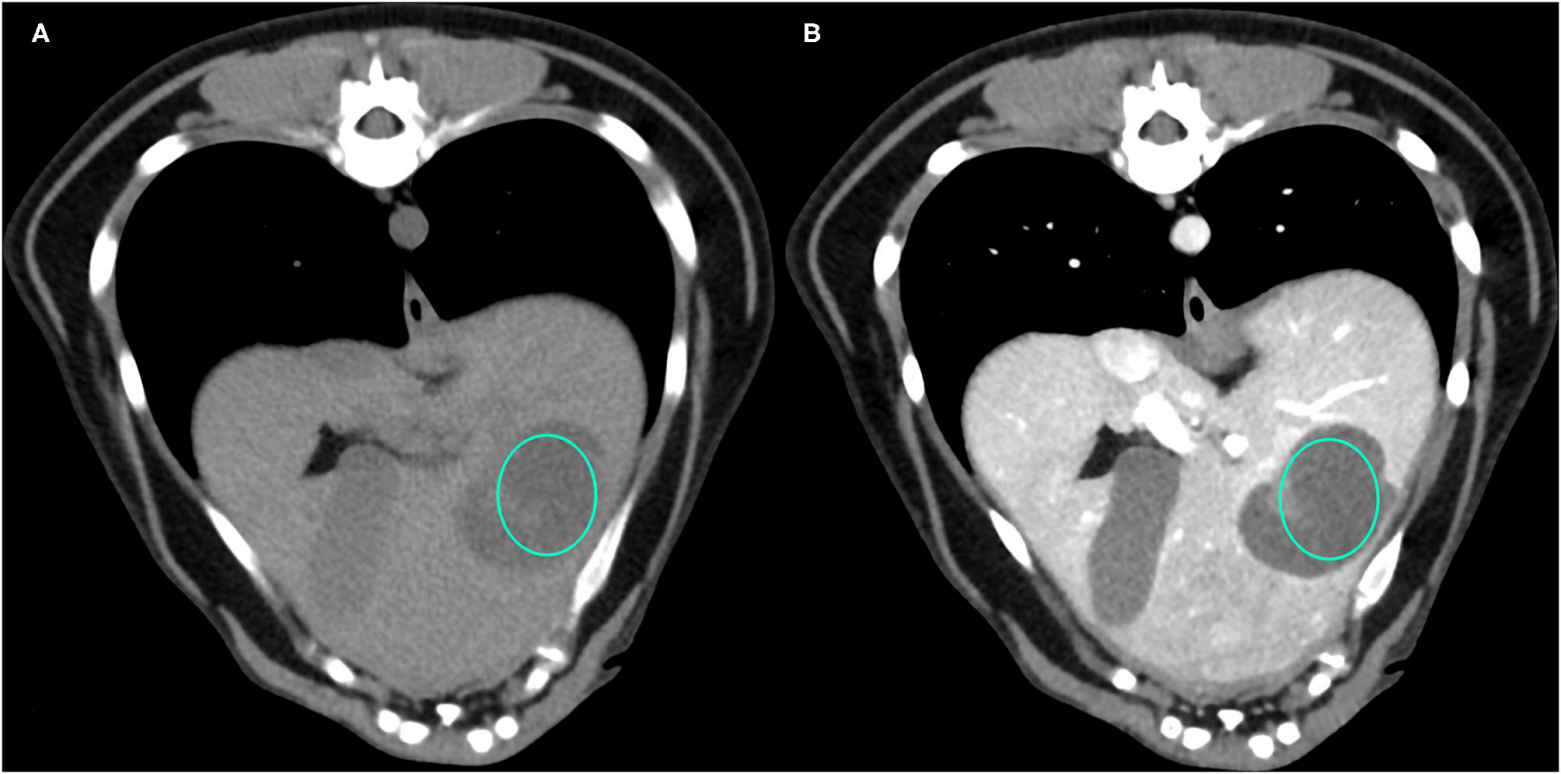
Example of a NH lesion that shows hypoattenuation and hypoenhancement, diffuse contrast enhancement pattern, with heterogeneous distribution, well-defined margins, irregular surface, and cyst-like appearance. **(A)** image obtained from the pre-contrast scan; **(B)** image obtained from the delayed scan. A ROI is placed inside the lesion in both. Based on the developed decision tree this lesion was classified as OBL.

**Figure 2:**
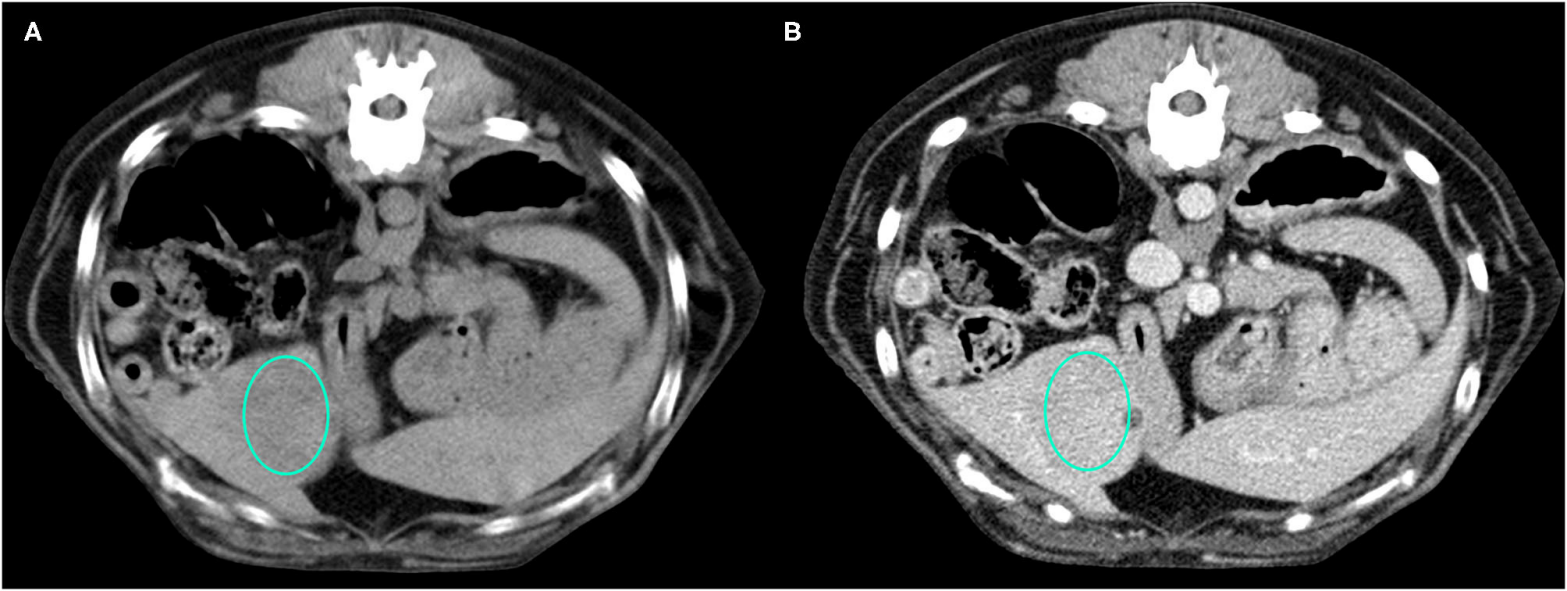
Example of an OBL lesion (diagnosed as adenoma) showing hyperattenuation and hypoenhancement, diffuse contrast enhancement pattern, with homogeneous distribution, well-defined margins, regular surface, and cyst-like appearance. **(A)** image obtained from the pre-contrast scan; **(B)** image obtained from the delayed scan. A ROI is placed inside the lesion in both. Based on the developed decision tree this lesion was classified as OBL.

**Figure 3:**
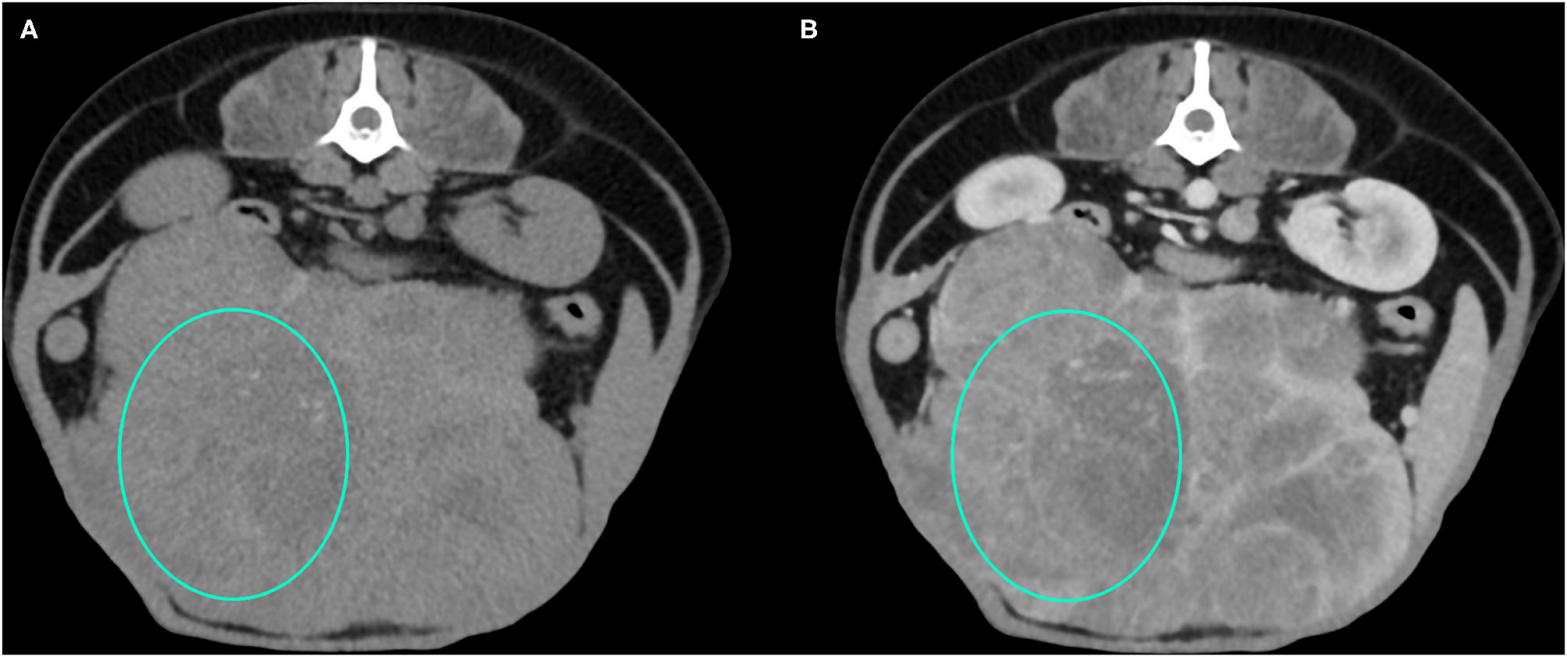
Example of an HCC showing hypoattenuation and hypoenhancement, diffuse contrast enhancement pattern, with heterogeneous distribution, well-defined margins, irregular surface, and cyst-like appearance. **(A)** image obtained from the pre-contrast scan; **(B)** image obtained from the delayed scan. A ROI is placed inside the lesion in both. Based on the developed decision tree this lesion was classified as HCC.

**Figure 4:**
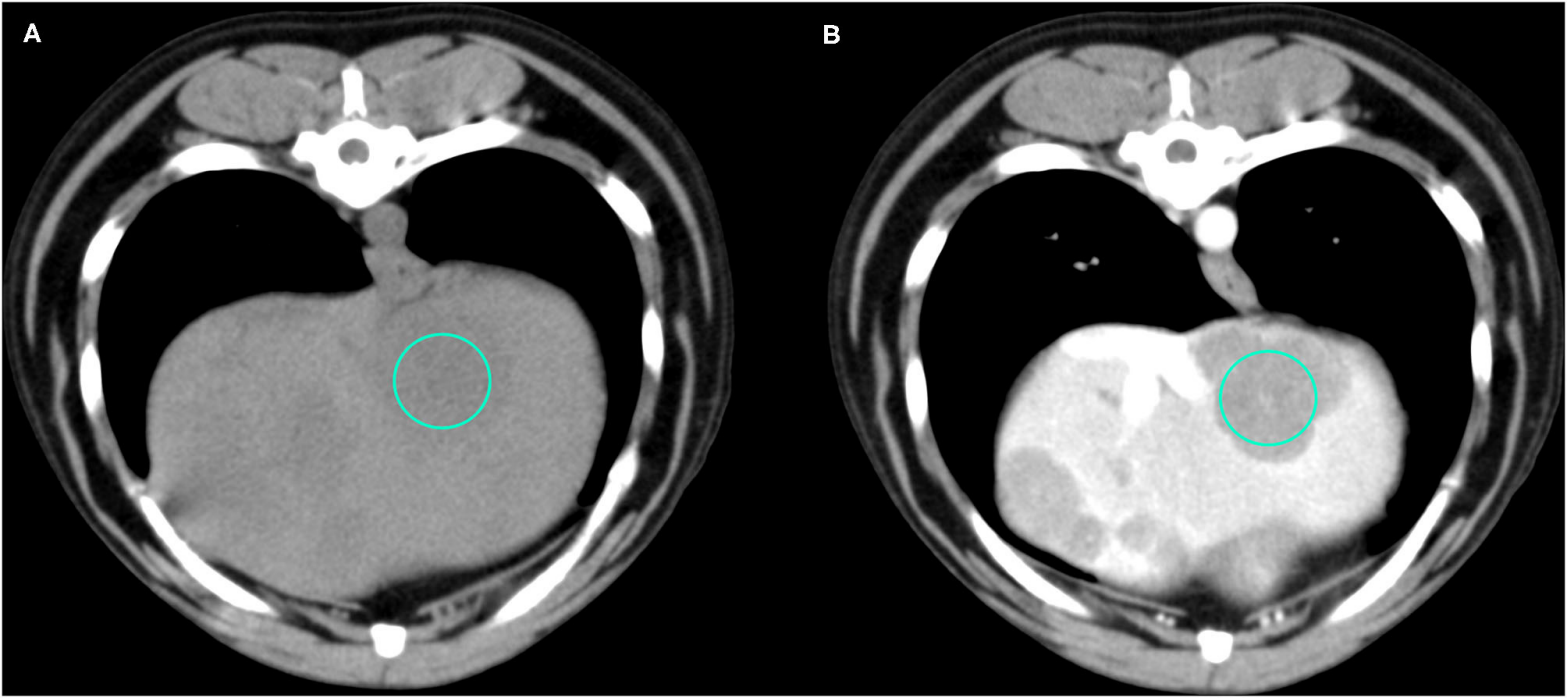
Example of an OML (diagnosed as lymphoma) showing hypoattenuation and hypoenhancement, diffuse contrast enhancement pattern, with heterogeneous distribution, well-defined margins, irregular surface, and solid appearance. **(A)** image obtained from the pre-contrast scan; **(B)** image obtained from the delayed scan. A ROI is placed inside the lesion in both. Based on the developed decision tree this lesion was classified as OML.

Representative cases of each FLL showing the most typical CT features are reported in Figures 1–4.

**Figure 5:**
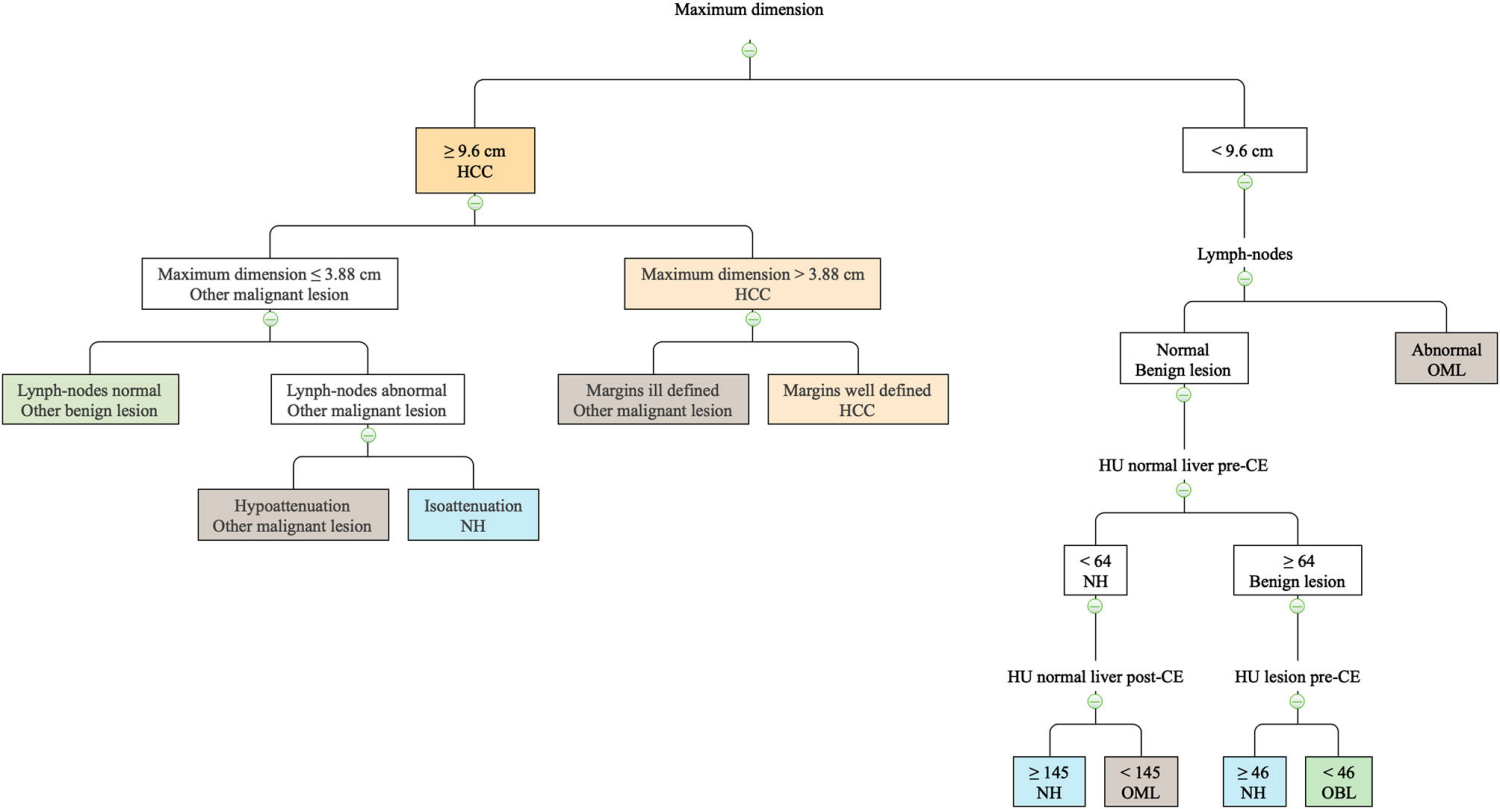
The machine learning-based decision tree developed based on qualitative and quantitative CT features of the FLLs.

The final decision tree algorithm was built on five automatically selected CT features: 1 qualitative feature (lymph nodes), and 4 quantitative features (maximum dimension, HU normal liver pre- contrast, HU normal liver post-contrast, HU lesion pre-contrast). The confusion matrix along with is reported in Table 3. The sensitivity, specificity, accuracy, precision, Mathews correlation coefficient, and the misclassification rate for each group, along with the global misclassification rate are reported in Table 4. The decision tree is set out in Figure 5.

**Table 3:** Confusion matrix to summarize the performance of the machine learning algorithm giving the number of predicted cases among the four categories of FLLs.

		**Actual**				
		**Nodular hyperplasia**	**Other benign lesion**	**Hepatocarcinoma**	**Other malignant lesion**	**Total**
Predicted	Nodular hyperplasia	10	3	2	4	19
	Other benign lesions	1	11	0	0	12
	Hepatocarcinoma	3	0	8	1	12
	Other malignant lesions	5	4	3	14	26
	Total	19	18	13	19	69

**Table 4:** Complete results of the classification of the FLLs based on the machine learning-based decision tree.

	**Nodular hyperplasia**	**Other benign lesions**	**Hepatocarcinoma**	**Other malignant lesions**
Sensitivity (%)	53	61	62	74
Specificity (%)	82	98	93	76
Accuracy (%)	74	88	87	75
Precision (%)	53	92	67	54
MCC^*^	0.42	0.69	0.58	0.51
Misclassification rate (%)	47	39	38	26
Global misclassification rate (%)	38			

* *MCC, Matthews Correlation Coefficient*.

The decision tree misclassified 7 cases as OBL and 5 cases as OML. Among the 7 incorrectly classified OBLs, 6 were in fact degenerations (misclassified as OML in 4 cases, and as NH in 2 cases). The remaining case classified as OBL was in fact a hepatocellular adenoma (misclassified as NH). Among the 5 incorrectly classified OMLs, 1 metastatic splenic sarcoma was misclassified as HCC. Lastly, 1 mast cell tumor, 1 endocrine neoplasia, 1 myeloma, and 1 metastasis of splenic sarcoma were classified as NH.

## Discussion

A machine learning-based, easy-to-follow, algorithm to predict the histotype of canine liver lesions, based on their CT features, is proposed. The proposed algorithm had a variable accuracy in the classification of the different histotypes, ranging from 0.74 of NH to 0.88 of OBL. On the other hand, also the precision was extremely variable ranging from 0.53 of NH to 0.92 of OBL. The global misclassification rate was high with 38% of the lesion that were incorrectly classified. The same machine learning algorithm (decision tree) used in this paper was used by the authors in a previous report (2) on the CEUS features of canine malignant liver lesions. In that report, a higher number of cases (185 total cases) was available, thus offering the scope to split the dataset into a training and a test set. In the present study, the relatively low number of available cases did not allow such a division of the dataset, and, therefore, the accuracy of the decision tree was retested on the same dataset.

A machine learning-based approach was used by other authors (13). In their report, Griebie et al. (13) used both a stepwise discriminant analysis and a Fisher prediction equation to identify the CT or ultrasound features that might be helpful in distinguishing between focal liver lesions. Using such a classification method allowed them to accurately (sensitivity = 96.7; specificity = 87.5) distinguish only between benign and malignant lesions. On the other hand, none of the features described by Griebie et al. (13) resulted as significant when a specific diagnosis classification was used.

In the present study, the different histotypes of the lesions were grouped into four different categories, in order to have a sufficient number of cases in each category. Using such a classification scheme and applying the decision tree enabled us to progressively detect those features that might be helpful in distinguishing between specific categories of lesion. On the other contrary, using such an approach results in an overall lower accuracy of the model when compared to the binary classification proposed by Griebie et al. (13).

A limitation of the present study is that, due to the fact that the masses were mostly incidental findings, only the delayed phase was evaluated in this study. In other studies on the same topic (4, 11, 13, 14, 26) the enhancement patterns have been evaluated in triple phase (arterial, portal, delayed). On the other hand, most (not all) of the CT features that are reported to be helpful in the distinction among different FLL are seen in the delayed phase. There is no agreement among different authors regarding the CT features of canine HCCs in the delayed phase. Indeed, Taniura et al. (4), and Fukushima et al. (10) describe HCCs as hypoenhancing lesions, Jones et al. (12) as hyperenhancing, and Kutara et al. (11) report that HCCs might have all the possible enhancement patterns. The results of the present study were similar to the findings by Taniura et al. (4) and Fukushima et al. (10). Indeed, all the HCCs included in this study were hypoenhancing lesions. Furthermore, HCCs have been described as being cyst-like lesions (10) with a heterogeneous contrast enhancement (11). These finding were also similar to those reported the present study; indeed, 12/13 HCCs were cyst-like lesions and a heterogeneous distribution of the contrast medium was always evident. On the other hand, as a result of the decision tree, the most effective CT feature to classify HCCs was the maximum dimension of the lesion. Interestingly, the cut-off value identified by the decision tree (9.6 cm) was very similar to the cut-off value reported by Griebie et al. (13) (9.5 cm) to identify malignant lesions.

The presence of an hyperenhancing fibrous capsule surrounding the lesion is reported to be a distinctive CT feature of HCCs in humans, and, therefore, is used in the distinction between HCCs and NHs (22). While this feature is distinctive in people, the feature was only present in 7/13 dogs with HCC and in none of the NHs in the current study. Furthermore, also Taniura et al. (4) and Fukushima et al. (10) identified a hyperenhancing capsule in 25/36 and 13/14 HCC cases, respectively. Lastly, the presence of a hyperenhancing capsule in NHs in dogs is reported only in a single case (10). Nevertheless, the presence of such a capsule was also evident in 7/18 OBLs and in 9/19 OMLs.

There is also no agreement among different authors regarding the CT features of NH. Indeed, they are described as isoenhancing lesions in the venous phase by Taniura et al. (4), and Fukushima et al. (10). On the contrary, Kutara et al. (11) describe them as often being hyperenhancing or isoenhancing lesions showing a homogeneous distribution of the contrast medium. Interestingly, in the present study, most of the NH were hypoenhancing lesions showing a diffuse enhancement pattern. In human patients, NHs are reported to be mainly isoenhancing during the venous phase (22).

A limitation of the present study is that Tru-cut biopsies were obtained only in 13 patients and only 3 patients had both cytology and histology. Tru-cut and incisional biopsies are, nowadays, considered the gold-standard diagnostic techniques to determine the histopathological subtype of FLLs (14, 27) and, indeed either Tru-cut (10, 13) or surgical biopsies (4, 11, 14) have been used as reference standard in previous studies. However, because of the possible side effects, such as hemorrhage, hypercoagulable states, hypotension, peritonitis, hepatic emphysema (28–30), biopsies are not always performed in patients with FLL. In these cases, cytological examination represents a viable alternative to biopsies, even if the sensitivity for malignancy is lower (31, 32). We are aware of the lack of standardization of the injection procedures and also that manual procedures are slightly operator-dependent; however, the key-point is that each subject of our study was scanned during an earlier or slightly more advanced stage of the delayed phase. We would like to state again that this is not a relevant hepatic phase study; in fact a specific arterial or portal phase was not performed in any subject. This study reports an analysis of the hepatic pathological CT patterns within slightly variable stages of the delayed phase that could be found during non-focused CT whole body examinations.

Another possible limitation of the present study is that the CT features of the lesions were evaluated only during the delayed phase. This was carried out because of the different technology of the CT scanners used to acquire the images for this study. Indeed, both 4- and 16-row CT scanners were used. The former does not enable images to be acquired during the arterial phase and, therefore, in order to make a reliable comparison, only the delayed phase was analyzed. At this point it is, however, important to stress that, in the study by Griebie et al. (13), using a multiphase CT scanner, only the CT features of the lesions in the venous and delayed phases were statistically significant for the development of their prediction model. Moreover, in a standard clinical protocol for CT total-body scan only the arterial and delayed phase are performed.

The decision tree, based on the qualitative and quantitative CT features of the lesions, reported in the present results could be an easy-to-use tool for the veterinary clinician in predicting the histotype of different canine FLLs. A larger number of cases, enabling application of stricter inclusion/exclusion criteria (for example using cut-off values for the dimensions of the lesions to be included) could, prospectively, enable creation of a more accurate decision tree. Nevertheless, as is often the case also in human medicine, the final histotype of a FLL should always be determined based on cytology or histology.

## Conclusions

The CT features of 69 FLL, analyzed in the pre-contrast and in the delayed phase, are reported. The developed machine learning algorithm had a 62% overall accuracy in the classification of the FLL based on their CT features. The misclassification rate was highest (47%) for NH and lowest for OML (26%). The use of the proposed decision tree, could, prospectively help the clinician in the evaluation of FLL.

## Data Availability Statement

The data analyzed in this study is subject to the following licenses/restrictions: Data are property of both the Department of Animal Medicine, Productions, and Health and of the Pedrani Veterinary Clinic. Requests to access these datasets should be directed to tommaso.banzato@unipd.it.

## Ethics Statement

Ethical review and approval was not required for the animal study because this is a retrospective clinical work and, therefore, no ethical approval is needed. Written informed consent was obtained from the owners for the participation of their animals in this study.

## Author Contributions

TB, SB, and AZ has conceived the study, performed the CT scans, drafted, and revised the manuscript. FB has revised the manuscript and revised part of the cytological examinations. BC drafted, revised the manuscript, and performed statistical analysis. All authors contributed to the article and approved the submitted version.

## Conflict of Interest

The authors declare that the research was conducted in the absence of any commercial or financial relationships that could be construed as a potential conflict of interest.
