# Corrigendum: Diagnostic Accuracy of Delayed Phase Post Contrast Computed Tomographic Images in the Diagnosis of Focal Liver Lesions in Dogs: 69 Cases

**DOI:** 10.3389/fvets.2021.782672

**Published:** 2021-11-04

**Authors:** Silvia Burti, Alessandro Zotti, Federico Bonsembiante, Barbara Contiero, Tommaso Banzato

**Affiliations:** ^1^Department of Animal Medicine, Productions and Health, University of Padua, Padua, Italy; ^2^Department of Comparative Biomedicine and Food Science, University of Padua, Padua, Italy

**Keywords:** decision tree, HCC (hepatic cellular carcinoma), contrast - enhanced CT, computed tomography, focal liver lesion

In the original article an error occurred with the ellipsoid volume formula and subsequently there were errors in Table 2. The volume was calculated using formula V = 4/3^*^(height^*^width^*^length) whereas the correct formula is: V = 4/3^*^(height/2^*^width/2^*^length/2). Therefore, the results reported in Table 2 are 8 times bigger than the actual volume. The corrected Table 2 appears below.

**Table 2:** Quantitative features of the lesions, classified based on cytological or histological examination, are reported as medians along with the first and third quartile values and the *p*-value.

	**HU normal liver pre-CE**	**HU normal liver post-CE**	**HU lesion pre-CE**	**HU lesion post-CE**	Max dimension^*^	Ellipsoid volume^**^
**DIAGNOSIS**						
Nodular hyperplasia (*n* = 19)	63.82 (53.79–69.79)^ab^	144.54 (120.59–169.15)	45.68 (40.72–54.79)	114.37 (50.96–144.87)	4.53 (2.45–6.75)^ab^	40.78 (6.15–112.86)^ab^
Other benign lesions^†^ (*n* = 18)	66.84 (64.36–72.54)^a^	137.60 (126.71–154.01)	39.50 (29.94–45.99)	75.65 (61.37–121.17)	2.15 (1.12–5.33)^b^	2.41 (0.39–26.78)^c^
Hepatocarcinoma (*n* = 13)	58.63 (53.12–63.02)^b^	127.72 (116.12–135.06)	41.48 (34.87–46.93)	67.39 (56.03–83.93)	11.11 (5.67–13.76)^a^	393.57 (54.80–727.31)^a^
Other malignant lesions (*n* = 18)	60.03 (54.59–64.55)^b^	142.87 (117.56–157.18)	39.93 (34.39–46.12)	83.19 (66.32–121.40)	3.59 (2.11–4.61)^b^	8.31 (3.67–23.60)^bc^
*p*-value	<0.01	0.29	0.80	0.13	<0.01	<0.01

*HU, Hounsfield Unit; c.m., contrast medium*.

* *Values are expressed in cm*.

** *Values are expressed in cm^3^*.

Different letters along columns means values significantly different for post-hoc multiple comparisons.

† *Other begin lesions = 1 biliary duct adenoma, 1 inflammation, 2 haematoma, 2 adenomas, 2 normal parenchyma, 11 degenerations*.

*‡Other malignant lesions = 1 mast cell tumor, 1 plasmocytoma, 1 biliary duct carcinoma, 1 undifferentiated carcinoma, 1 melanoma, 1 metastasis of mammary neoplasia, 2 lymphomas, 4 endocrine neoplasia, 7 sarcomas*.

The authors apologize for this error and state that this does not change the scientific conclusions of the article in any way. The original article has been updated.

## Publisher's Note

All claims expressed in this article are solely those of the authors and do not necessarily represent those of their affiliated organizations, or those of the publisher, the editors and the reviewers. Any product that may be evaluated in this article, or claim that may be made by its manufacturer, is not guaranteed or endorsed by the publisher.

